# Commentary: Overall Survival in Heart Disease–Related Death in Non-Small Cell Lung Cancer Patients: Nonimmunotherapy Versus Immunotherapy Era: Population-Based Study

**DOI:** 10.3389/fonc.2021.639042

**Published:** 2021-04-22

**Authors:** Yiping Jiao, Chengqi Qian, Shumin Fei

**Affiliations:** ^1^School of Automation, Southeast University, Nanjing, China; ^2^Jiangsu Chunyu Education Group Co., Ltd., Nanjing, China

**Keywords:** immunotherapy, non-small cell lung cancer, heart diseases, adverse effects, survival analysis

## Introduction

Immunotherapy has been advised for advanced-stage non-small-cell lung cancer (NSCLC) patients; however, it could lead to heart disease-related death (HDD) due to cardiovascular toxicity. A recent study (1) compared the overall survival of HDD patients in the immunotherapy era with the non-immunotherapy era in the Surveillance, Epidemiology, and End Results (SEER) dataset. Specifically, advanced-stage NSCLC death cases in 2015 and 2007 were used as a study cohort. By comparing the death cases, it concluded that the HDD risk is significantly higher in the immunotherapy era, with a hazard ratio (HR) of 1.31, 95% CI: 1.099–1.570. Pericardial effusion is sometimes reported in NSCLC patients who receive immunotherapy (2). In very rare cases, immunotherapy could lead to autoimmune myocarditis (3). However, we believe that the HDD risk is overestimated in the commented study. We argue that using the death cases only could lead to potential issues; for example, the differences in age distribution and life span may mislead the conclusions. In this commentary, we reconsidered this problem; however, we found that the HDD risk in the immunotherapy era is actually lower than that in the counterpart by considering the whole advanced-stage NSCLC patient cohort.

## Methods and Results

We would argue that the whole advanced-stage NSCLC patients (referred to as a “cohort”) should be considered in the evaluation, rather than focusing on the subtle pattern among cases of confirmed death. For this, the following are considered:

1. There are 243,637 incidences of advanced-stage (IIIB and IV) NSCLC collected in the SEER dataset from 2000 to 2017.
2. Kaplan–Meier (K-M) analysis for HDD risk in the cohort is carried out. Moreover, the competing risk model is also considered in order to avoid overestimating the risk (4).
3. Data on the underlying cause of death in WONDER (5) are collected from 1999 to 2018. We compared the HDD risk in the cohort with that in the general public.

### Survival Analyses

**Figure 1:**
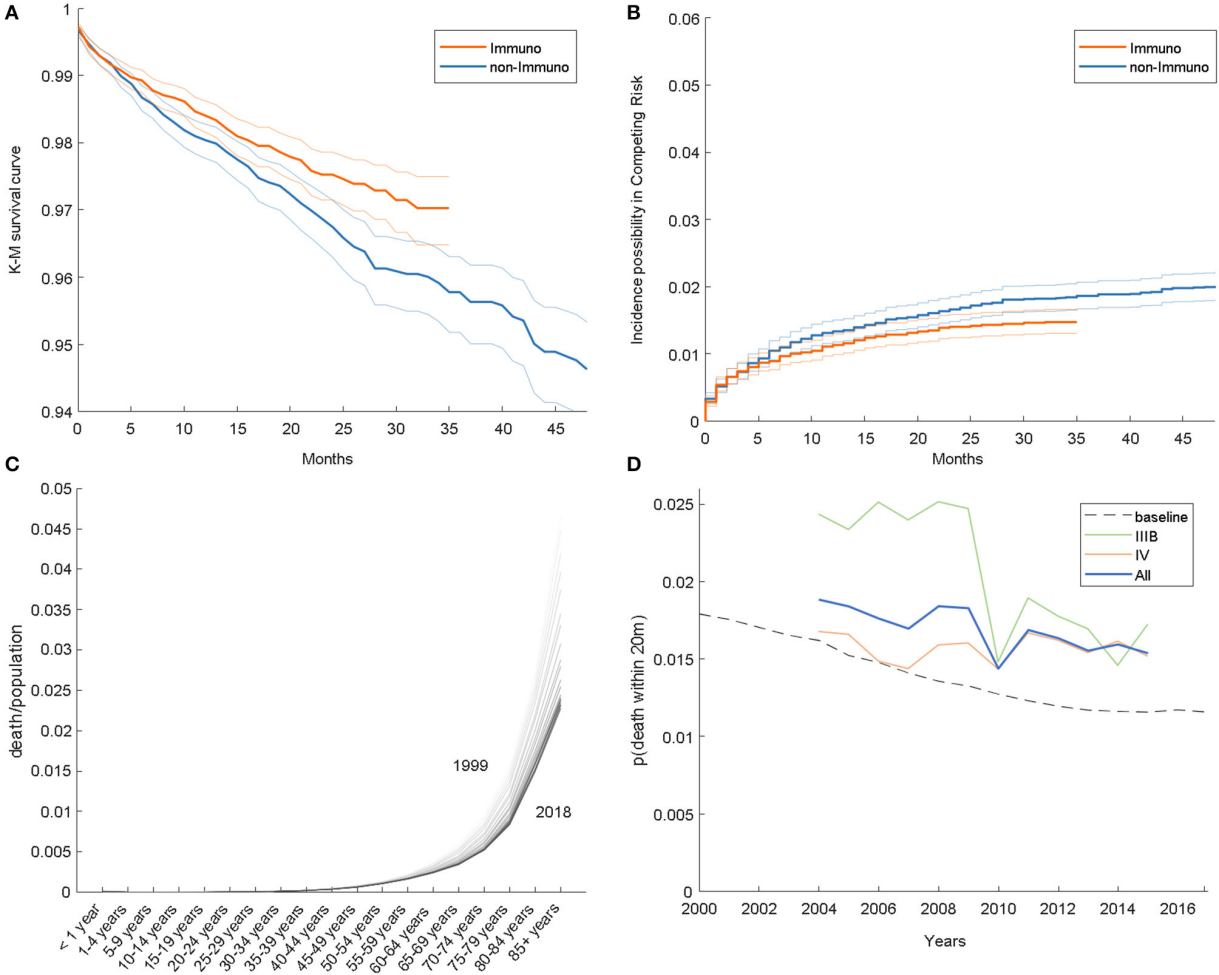
**(A)** Kaplan–Meier survival curves. **(B)** Estimated incidence possibility in the competing risk model. **(C)** Age-specific HDD risk in the general population. **(D)** HDD event frequency in the SEER dataset.

In accordance with the previous study, we compared the HDD event between 2007 and 2015; the results of the K-M analysis and competing risk model are given in Figures 1A,B, respectively. At 20 months, the HDD survival ratios of the immunotherapy and non-immunotherapy groups in the K-M analysis are 97.79 and 97.23%, respectively. In the competing risk model, the estimated incidences of HDD are 1.34 and 1.57% in the two groups. From these results, we demonstrated that the HDD risk in the immunotherapy group is lower than that in the counterpart, with a relative hazard ratio of 0.8535 and 0.7978 from K-M and the competing risk model, respectively.

### Comparison With the General Population

It would be interesting to know whether lung cancer itself is correlated with heart disease risk. For this, we calculated the frequency of the event (heart disease-related death within 20 months after NSCLC diagnosis), shown as a solid curve in Figure 1D; the risk in 2015 is actually lower than in 2007, with a relative ratio of 0.718, 1.055, and 0.906 for IIIB, IV, and both stages, respectively. Overall, the frequency of HDD is decreasing among years, which is consistent with Figures 1A,B.

Furthermore, we analyzed HDD data of the general population from WONDER, illustrated in Figure 1C. As shown, the mortality rises as the age becomes older; on the other hand, the overall curve is descending over time. The age distribution of the cohort also varies among years; for example, the cohort is composed of more individuals of age above 55 in 2015 than in 2007 (89.1 vs. 91.9%). To consider these aspects jointly, we estimated a risk baseline for the cohort, which is synthesized by weighting the general HDD risk by the age distribution. The baseline is shown as a dashed line in Figure 1D. As shown, the HDD risk in advanced-stage NSCLC patients is higher than that in the general population. A similar phenomenon was also reported in other types of cancer, for example, endometrial cancer (6).

## Conclusion

In this commentary, we reconsidered the HDD risk in advanced-stage NSCLC patients who receive immunotherapy. From the perspective of K-M analysis, competing risk model, and frequency, we found that the HDD risk is decreasing among years. Based on these findings, we argue that there is no sufficient evidence that immunotherapy can lead to a significantly higher HDD risk in NSCLC patients. Nevertheless, the NSCLC patients indeed have a higher HDD risk than the general population, for which further investigation is needed.

## Author Contributions

YJ collected and analyzed the data, finished the initial version of the manuscript. CQ finished the survival analysis. SF went through the paper. All the authors participated in the writing and reviewing of the paper.

## Conflict of Interest

CQ was employed by Jiangsu Chunyu Education Group Co., Ltd. The remaining authors declare that the research was conducted in the absence of any commercial or financial relationships that could be construed as a potential conflict of interest.
